# Genome Sequence of the Freshwater Yangtze Finless Porpoise

**DOI:** 10.3390/genes9040213

**Published:** 2018-04-16

**Authors:** Yuan Yuan, Peijun Zhang, Kun Wang, Mingzhong Liu, Jing Li, Jinsong Zheng, Ding Wang, Wenjie Xu, Mingli Lin, Lijun Dong, Chenglong Zhu, Qiang Qiu, Songhai Li

**Affiliations:** 1 Center for Ecological and Environmental Sciences, Northwestern Polytechnical University, Xi’an 710072, China; 2017203291@mail.nwpu.edu.cn (Y.Y.); wk8910@gmail.com (K.W.); nwpulijing@163.com (J.L.); 2014303644@mail.nwpu.edu.cn (W.X.); Long9602@mail.nwpu.edu.cn (C.Z.); 2 Qingdao Research Institute, Northwestern Polytechnical University, Qingdao 266200, China; 3 Marine Mammal and Marine Bioacoustics Laboratory, Institute of Deep-sea Science and Engineering, Chinese Academy of Sciences, Sanya 572000, China; pjzhang@idsse.ac.cn (P.Z.); liumingzhong@idsse.ac.cn (M.L.); mingli@idsse.ac.cn (M.L.); donglj@idsse.ac.cn (L.D.); 4 Institute of Hydrobiology, Chinese Academy of Sciences, Wuhan 430072, China; zhengjinsong@ihb.ac.cn (J.Z.); wangd@ihb.ac.cn (D.W.)

**Keywords:** Yangtze finless porpoise, endangered species, genome, genome assembly, annotation, genome evolution

## Abstract

The Yangtze finless porpoise (*Neophocaena asiaeorientalis* ssp. *asiaeorientalis*) is a subspecies of the narrow-ridged finless porpoise (*N. asiaeorientalis*). In total, 714.28 gigabases (Gb) of raw reads were generated by whole-genome sequencing of the Yangtze finless porpoise, using an Illumina HiSeq 2000 platform. After filtering the low-quality and duplicated reads, we assembled a draft genome of 2.22 Gb, with contig N50 and scaffold N50 values of 46.69 kilobases (kb) and 1.71 megabases (Mb), respectively. We identified 887.63 Mb of repetitive sequences and predicted 18,479 protein-coding genes in the assembled genome. The phylogenetic tree showed a relationship between the Yangtze finless porpoise and the Yangtze River dolphin, which diverged approximately 20.84 million years ago. In comparisons with the genomes of 10 other mammals, we detected 44 species-specific gene families, 164 expanded gene families, and 313 positively selected genes in the Yangtze finless porpoise genome. The assembled genome sequence and underlying sequence data are available at the National Center for Biotechnology Information under BioProject accession number PRJNA433603.

## 1. Introduction

**Figure 1:**
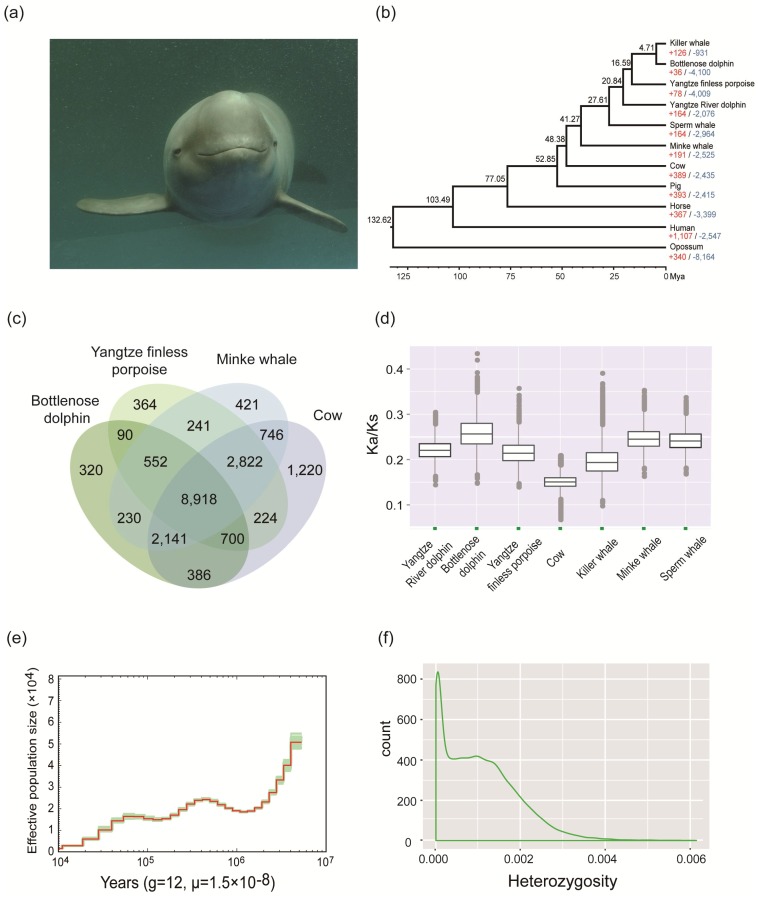
Gene families, phylogenetic relationships, and demographic history of the Yangtze finless porpoise. (**a**) Picture of a Yangtze finless porpoise (image from SL); (**b**) Phylogenetic tree constructed using the maximum likelihood approach and a comparison of gene family numbers. Black numbers next to the branches indicate divergence times, while the red and blue numbers indicate the number of gene families that have expanded or contracted, respectively, since the split from the common ancestor; (**c**) Venn diagram showing unique and overlapping gene families in the Yangtze finless porpoise, common minke whale, bottlenose dolphin, and cow genomes. Each number represents a gene family number; (**d**) Box-plot showing ratios of non-synonymous to synonymous mutations (Ka/Ks) in the Yangtze finless porpoise, Yangtze River dolphin, bottlenose dolphin, cow, killer whale, common minke whale, and sperm whale genomes; (**e**) Demographic history of the Yangtze finless porpoise constructed using the pairwise sequentially Markovian coalescence model; (**f**) Distribution of heterozygosity in the Yangtze finless porpoise genome (heterozygosity ratios of non-overlapping 50 K windows).

The Yangtze finless porpoise (*Neophocaena asiaeorientalis* ssp. *asiaeorientalis*) is a subspecies of the narrow-ridged finless porpoise (*N*. *asiaeorientalis*). Nicknamed the ‘panda in water’, it occurs solely in the middle and lower reaches of the Yangtze River and its adjunct lakes and tributaries [1]. The Yangtze finless porpoise is one of the smallest cetaceans [2] and is a flagship species for conservation of the freshwater ecological system in the Yangtze River. Its habitat overlaps that of the Yangtze River dolphin (baiji, *Lipotes vexillifer*), which was recognized as functionally extinct in 2006 [3], and it is therefore suffering from the same environmental survival pressure. Compared with the Yangtze River dolphin, the Yangtze finless porpoise prefers to interact with humans and is potentially more vulnerable to the adverse effects of human activities. Following the likely extinction of the Yangtze River dolphin, it is now the only cetacean living in the Yangtze River (Figure 1a) [3]. A series of studies revealed an accelerated population decline of the Yangtze finless porpoise since the early 1990s, with the population in the main stream of the Yangtze River between Yichang and Shanghai declining from more than 2500 in 1991 [4] to 1225 in 2006 [5], and to 505 in 2012 [6]. The current total population of the Yangtze finless porpoise, including those in Poyang and Dongting Lakes, has been estimated to be about 1000 [6]. The Yangtze finless porpoise is now at extremely high risk of extinction in the next 100 years [7]. Therefore, it is listed as critically endangered in the International Union for Conservation of Nature and Natural Resources Red List [8] and appendices of both the Convention on the Conservation of Migratory Species of Wild Animals and the Convention on International Trade in Endangered Species of Wild Fauna and Flora [9].

The Yangtze finless porpoise is the only freshwater species in the porpoise family [1]. It may have unique adaptions in the porpoise family and cetacean lineage. While the morphology of the Yangtze finless porpoise has been studied intensively because of its unique features [10,11], the underlying genetics and its evolution have received much less attention. Genomic information is imperative for understanding the evolution and adaptation of the Yangtze finless porpoise.

Here, we report the first sequencing, assembly, and annotation of the Yangtze finless porpoise genome. Our comparative genomic analysis provides insights into its freshwater adaptation and was used to reconstruct the demographic history of the Yangtze finless porpoise. Our results might also shed light on effective methods for conserving the endangered finless porpoise.

## 2. Materials and Methods, Results, and Discussion 

Genomic DNA was isolated from the muscle tissue of an adult female Yangtze finless porpoise that died accidentally on 28 October 2010 in Tian-e-Zhou Baiji National Natural Reserve, Hubei, China, in a capture and release scenario for regular medical examination and population investigation of porpoises in the reserve. Sample collection and use protocols were approved by the Institute of Deep-sea Science and Engineering, Chinese Academy of Sciences, with the ethics approval code SIDSSE-SYLL-MMMBL-01. Using a whole genome shotgun sequencing strategy, we constructed four DNA paired-end libraries of 289, 462, 624, and 791 base pairs (bp) and mate-paired libraries of 4, 7, 11, and 18 kb, which were sequenced using an Illumina HiSeq 2000 platform with 150 bp read lengths (Table S1). In total, 714.28 Gb of raw sequence reads were generated. We subsequently filtered these raw reads using the SoapFilter v2.2 [12] software to remove reads with >10% unknown bases, paired reads with 50% low-quality bases (quality scores ≤5), and reads with PCR duplicates or adapter contamination. This left 581.59 Gb of clean sequence data in total. Then, we corrected the short-insert library reads using k-mer-based correction with the Lighter v1.1.1 software [13]. Finally, 580.28 Gb of corrected clean sequence data were retrieved for assembly.

We used all of the cleaned reads from paired-end libraries to estimate the genome size of the Yangtze finless porpoise on the basis of k-mer analyses with the following formula: 

G = k-mer_number/k-mer_depth [14]. In total, 211,733,348,694 k-mers were generated, with a peak k-mer depth of 85 (Table S2). The estimated genome size is approximately 2.49 Gb (Figure S1), which is slightly shorter than the genomes of the Yangtze River dolphin (2.84 Gb) [15] and the common minke whale (*Balaenoptera acutorostrata*) (2.76 Gb) [16].

The Platanus v1.2.4 [17] software was used for the whole assembly procedure, which was divided into three parts: contig assembly, scaffold construction, and gap closure. In the first step, we used the short-insert reads to construct *de Bruijn* graphs, which were assembled into distinct contigs with default parameters. Then, we constructed scaffolds with cleaned paired-end and mate-paired reads based on the information. Finally, in the gap-closure step, we used reads mapped on scaffolds to fill the gaps. The final size of the Yangtze finless porpoise genome assembly was 2.22 Gb, approximately 89.16% of the estimated genome size, with contig and scaffold N50 values of 46.69 kb and 1.71 Mb, respectively (Table S3).

Next, we used the benchmarking universal single-copy orthologs (BUSCO, v3.0) [18] software package and mammalia_odb9 gene set, which contains 4104 single-copy genes that are highly conserved in mammals, to assess the completeness of the assembly. We obtained a 92.8% BUSCO completeness value (with 92% and 0.8% of the 4104 genes detected as single copies and duplicates, respectively, 3.2% fragmented, and 4.0% missing) (Table S4). The results indicate that the Yangtze finless porpoise genome assembly has high completeness. The sequencing reads from pair-end libraries were aligned to our genome assembly with the Burrows–Wheeler Aligner (BWA) software [19], and more than 99% of the genome had >20-fold coverage (Figure S2).

Repetitive regions of the Yangtze finless porpoise genome were identified with a combination of de novo prediction and homolog searches. First, for de novo predictions, we constructed a de novo repeat library with RepeatModeler (v1.0.8, http://www.repeatmasker.org/RepeatModeler) and LTR_FINDER [20]. Then, we used RepeatMasker v3.3.0 [21] to detect additional repeats in the sequences. To search for homologs, we identified tandem repeats in our draft genome with Tandem Repeats Finder v4.07. We also searched for transposable elements (TEs), using RepeatMasker v4.0.5 and RepeatProteinMask (v3.3.0, a package in RepeatMasker) with the default parameters, to detect matches in the Repbase and TE protein databases. The combined results of these methods indicated that repeat sequences accounted for 39.98% of the Yangtze finless porpoise genome, and long interspersed elements were predominant in the repetitive regions (Tables S5 and S6).

We also used de novo prediction and homology-based searches to identify protein-coding genes. For homology-based gene prediction, protein sequences from the cow (UMD3.1) and five cetaceans (killer whale, *Orcinus orca* [22], Yangtze River dolphin [15], common minke whale [16], sperm whale, *Physeter macrocephalus* [23], and bottlenose dolphin, *Tursiops truncatus* [22]) (Table S7) were aligned to the repeat-masked Yangtze finless porpoise genome with tBLASTN [24]. Then, we used Exonerate v2.2 [25] to filter the genome sequences and the corresponding query proteins and search for accurately spliced alignments. For de novo annotation, Augustus v3.2.1 [26], GeneID v1.4.4 [27], and GlimmerHMM v3.0.3 [28] were used to predict genes within the genome on the basis of a human training set. Next, we used EVidenceModeler v1.1.1 [29] to integrate homologs and de novo predicted genes and generate a comprehensive, non-redundant gene set (Table S8). After filtering short low-quality genes (encoding proteins with <50 amino acids) exhibiting premature termination, 18,479 genes were predicted in the Yangtze finless porpoise genome, and the number of genes, gene length distribution, and exon number per gene were similar to those of other mammals (Table S9 and Figure S3). We also identified 2667 pseudogenes in the genome (Table S10). 

The protein sequences predicted from the Yangtze finless porpoise genome were aligned with entries in the Swiss-Prot and TrEMBL databases with E-values < 1 × 10^−5^ using Ghostz [30]. We used InterProScan v5.25-64.0 to annotate detected motifs and domains by searching public databases (Pfam, ProDom, SMART, PRINTS, and PANTHER), and the Kyoto Encyclopedia of Genes and Genomes database to search for significantly enriched biological pathways. Approximately 99.45% of all of the predicted genes were annotated (Table S11).

To predict the species-specific genes in the Yangtze finless porpoise and genes shared with other species, we downloaded the protein sequences of 10 additional species (human, pig, horse, cow, opossum, killer whale, common minke whale, sperm whale, bottlenose dolphin, and Yangtze River dolphin) from the NCBI (National Center for Biotechnology Information, http://www.ncbi.nlm.nih.gov) and Ensembl databases (Table S7) [31]. Consensus gene sets for the additional species were filtered to keep the longest coding sequence for each gene, removing those with premature stop codons or protein sequence lengths of less than 50 amino acids. We then applied an all-to-all blastp [24] strategy with an E-value of 1 × 10^−5^ and Markov chain clustering applied in OrthoMCL [32] with the default inflation parameter to define clusters of orthologous genes (Table S12). A total of 13,911 homologous gene families were identified, and 364 gene families were specific to the Yangtze finless porpoise compared with the cow, bottlenose dolphin, and common minke whale (Figure 1c). The unique gene families were significantly enriched in eight gene ontology (GO) terms (Table S13), and their functions were mainly associated with ion transport, including “sodium channel activity” (GO:0005272), “voltage-gated sodium channel activity” (GO:0005248), and “sodium channel complex” (GO:0034706). Using Computational Analysis of gene Family Evolution (CAFÉ, v4.0.1) [33] to identify signs of expansion and contraction of gene families, we detected 78 gene families that have apparently expanded in the Yangtze finless porpoise lineage (Figure S3). The expanded gene families were significantly enriched in 19 GO categories, and their functions were mainly related to cell adhesion and biological transport (Table S14).

Next, we selected 2619 single-copy gene families from the above 11 species and aligned the coding sequences from each single-copy family using PRANK v3.8.31 [34] with the codon option. Following this, we extracted four-fold degenerate sites from the single-copy genes, selected the GTR + G + I model, and used RAxML v7.2.8 [35] (Figure S4) to construct a phylogenetic tree. Finally, we applied the program BEAST [36] with the Bayesian approach and calibration against opossum/human, human/cow, cow/pig, minke whale/cow, and minke whale/sperm whale divergence times (124.6–134.8, 95.3–113, 48.3–53.5, 53.0–59.0, and 30.6–35.5 million years ago (Mya), respectively) [37] to estimate the divergence time of each node. Our phylogenetic results indicate that the Yangtze finless porpoise is closely related to the bottlenose dolphin and killer whale with a divergence time of approximately 16.59 Mya, and to the Yangtze River dolphin with a divergence time of approximately 20.84 Mya (Figure 1b).

We identified 7243 shared single-copy genes in the Yangtze finless porpoise, Yangtze River dolphin, killer whale, common minke whale, bottlenose dolphin, sperm whale, and cow genomes. We subsequently used gBlocks [38] to trim a multiple sequence alignment generated by PRANK, discarding alignments shorter than 150 bp. Next, we applied the program codeml in the PAML [39] package to estimate the average nonsynonymous to synonymous mutation (dN/dS) ratios with the free ratio model, and the branch-site likelihood ratio test to identify positively selected genes (PSGs) in the above seven species. We found that the Yangtze finless porpoise has an intermediate dN/dS value in comparison with the genomes of other mammal species (Figure 1d). Investigating PSGs in the Yangtze finless porpoise genome will provide insights into aquatic and freshwater adaptation. A total of 313 PSGs (Tables S14 and S15) were found in the Yangtze finless porpoise lineage. Our analysis revealed that several PSGs are associated with osmotic adjustment, including aquaporin 4 (*AQP4*), cystic fibrosis transmembrane conductance regulator (*CFTR*), and guanylate cyclase activator 2B (*GUCA2B*) [40,41,42]. *AQP4* encodes a member of the aquaporin family of intrinsic membrane proteins, which regulates body water balance. *CFTR* is associated with ion and water secretion and absorption in epithelial tissues, and *GUCA2B* encodes a preproprotein that binds to cognate receptors and may regulate salt and water homeostasis in the intestine and kidneys. In addition to genes related to osmotic adjustment, several candidate PSGs associated with DNA repair were also found, including *RTBDN*, *RAD18*, *RAD17*, and *FANCL* [43,44]. This could be relevant to the potentially stronger ultraviolet radiation (UVR) in freshwater environments compared with coastal seawater environments. Compared with coastal seawater, freshwater is more limpid and may be exposed to more UVR [45].

We also detected 171 GO categories [46] that have apparently evolved more rapidly in the Yangtze finless porpoise lineage than in other cetaceans (Table S16). These were mainly related to three functional groups potentially associated with freshwater adaptation. The first functional group is related to basic physiological activities and linked to the GO categories “oxidoreductase activity”, “ATPase activity”, and “metabolic process”. The second functional group is immune processes, including the GO categories “immune response”, “immune system process”, and “G-protein coupled receptor activity”, which has high presumed importance for adaptation to complex freshwater environments. During the switch from seawater to freshwater, the environmental pathogenic microorganisms changed dramatically for the Yangtze finless porpoise, and rapid immune system evolution might be important for this species [47]. The most prominent and important functional group was related to ion transmembrane transport, associated with the GO categories “potassium ion transmembrane transporter activity”, “transmembrane transporter activity”, and “transmembrane signaling receptor activity”. The balance of water and salt was the main challenge faced by the Yangtze finless porpoise during the transition from a hyperosmotic marine environment to a low-permeability freshwater environment. The Yangtze finless porpoise had to maintain its internal osmotic pressure balance by enhancing or changing transmembrane-related genes [48]. Consequently, additional functional and physiological experiments are needed to verify the contributions of the identified genes to freshwater adaptation. 

To elucidate the demographic history of the Yangtze finless porpoise further, we first used SAMtools v1.3.1 [49] to obtain a consensus genome sequence and divided it into 100 non-overlapping bins. Then, we used the pairwise sequentially Markovian coalescence (PSMC) model [50] with N25 -t15 -r5 -p ‘4 + 25 × 2 + 4 + 6’ parameters and bootstrapping (randomly sampling 100 times to estimate the variance of the effective population size). PSMC analysis generated a well-defined demographic history from 3,000,000 to 10,000 years ago (Kya). The effective population size of the Yangtze finless porpoise apparently declined around 3 Mya, remained stable between 1 Mya and 10 Kya, and declined steadily after 10 Kya (Figure 1e).

In total, 2.30 million single nucleotide variants (Table S17) and 2.03 million insertions and deletions (Table S18) were identified with SAMtools v1.3.1 following a strict quality control and then annotated with SnpEff v4.30 [51]. The estimated nucleotide heterozygosity was 0.10%, which is lower than the reported heterozygosity of the bottlenose dolphin (0.14%) [16]. Further analysis of the heterozygosity ratios in non-overlapping 50 K windows (Figure 1f) showed that regions with low ratios (<0.0003) accounted for a high proportion (26.13%) of the total. This is consistent with patterns observed in other endangered species [37] and is likely due to recent inbreeding in the Yangtze finless porpoise lineage linked to its small population.

In summary, we generated and analyzed a draft genome assembly of the Yangtze finless porpoise. We also reconstructed the demographic history of the Yangtze finless porpoise. The novel genome data will provide a valuable resource for cetacean research. The acquired data should facilitate further studies of the genetic basis of adaptations of this unique freshwater porpoise, of its conservation, and of the molecular differences between freshwater, marine, and terrestrial mammals.

## Supplementary Material

The following are available online at http://www.mdpi.com/2073-4425/9/4/213/s1, Figure S1: 17-mer distribution in the Yangtze finless porpoise genome, Figure S2: Sequence depth distribution of the assembly data, Figure S3: Comparison of gene structure characteristics of Yangtze finless porpoise and other cetaceans, Figure S4: Phylogeny relationships between the Yangtze finless porpoise and other mammals reconstructed by RAxML with the GTR + G + I model; Tables S1: Summary of sequenced reads, Table S2: 17-mer depth distribution, Table S3: Statistics for the final assemblies of the Yangtze finless porpoise genome, Table S4: Summary of BUSCO analysis of matches to the 4,104 mammalian BUSCOs, Table S5: Prediction of repetitive elements in the assembled Yangtze finless porpoise genome, Table S6: Summary statistics of interspersed repeat regions, Table S7: Data on all species used during the genome analysis, Table S8: Prediction of protein-coding genes in the Yangtze finless porpoise, Table S9: Summary statistics of comparative gene structure, Table S10: Summary of the predicted pseudogenes, Table S11: Functional annotation of predicted genes in the Yangtze finless porpoise genome, Table S12: Summary statistics of gene families in 11 species, Table S13: GO enrichment analysis of the unique gene families in the Yangtze finless porpoise lineage, Table S14: GO enrichment analysis of the expanded gene families in the Yangtze finless porpoise lineage, Table S15: Candidate PSGs in the Yangtze finless porpoise lineage, Table S16: The classification of the candidate PSGs, Table S17: GO categories showing accelerated evolutionary rates in the Yangtze finless porpoise lineage and the other cetaceans.
