# Supplementary material for: Genome Sequence of the Freshwater Yangtze Finless Porpoise
Source: Genes (Basel). 2018 Apr 16;9(4):213. doi: 10.3390/genes9040213 (PMC5924555; doi:10.3390/genes9040213)
Supplement: Supplementary file 1 [file genes-09-00213-s001.pdf]

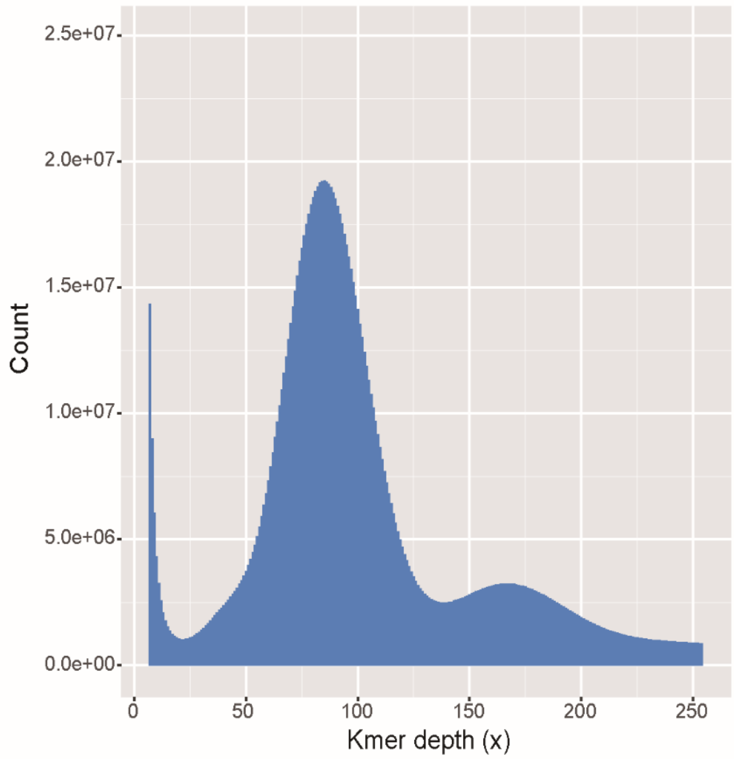

Figure S1. 17-mer distribution in the Yangtze finless porpoise genome. The x-axis is 17-mer depth (X); the y-axis is the number of sequencing reads at that depth.

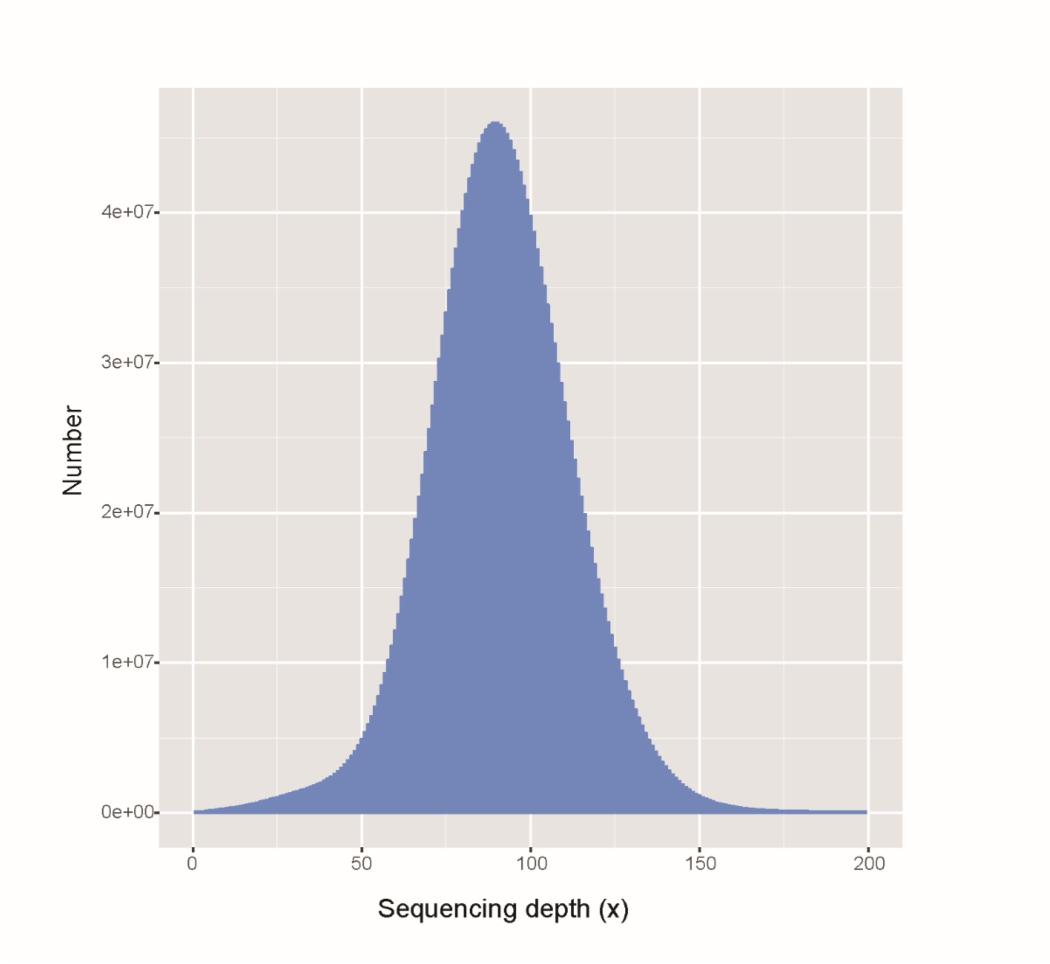

Figure S2. Sequence depth distribution of the assembly data. The x-axis shows the sequencing depth (X) and the y-axis shows the number of bases at a given depth. The results demonstrate that 99% of bases sequencing depth is more than 20.

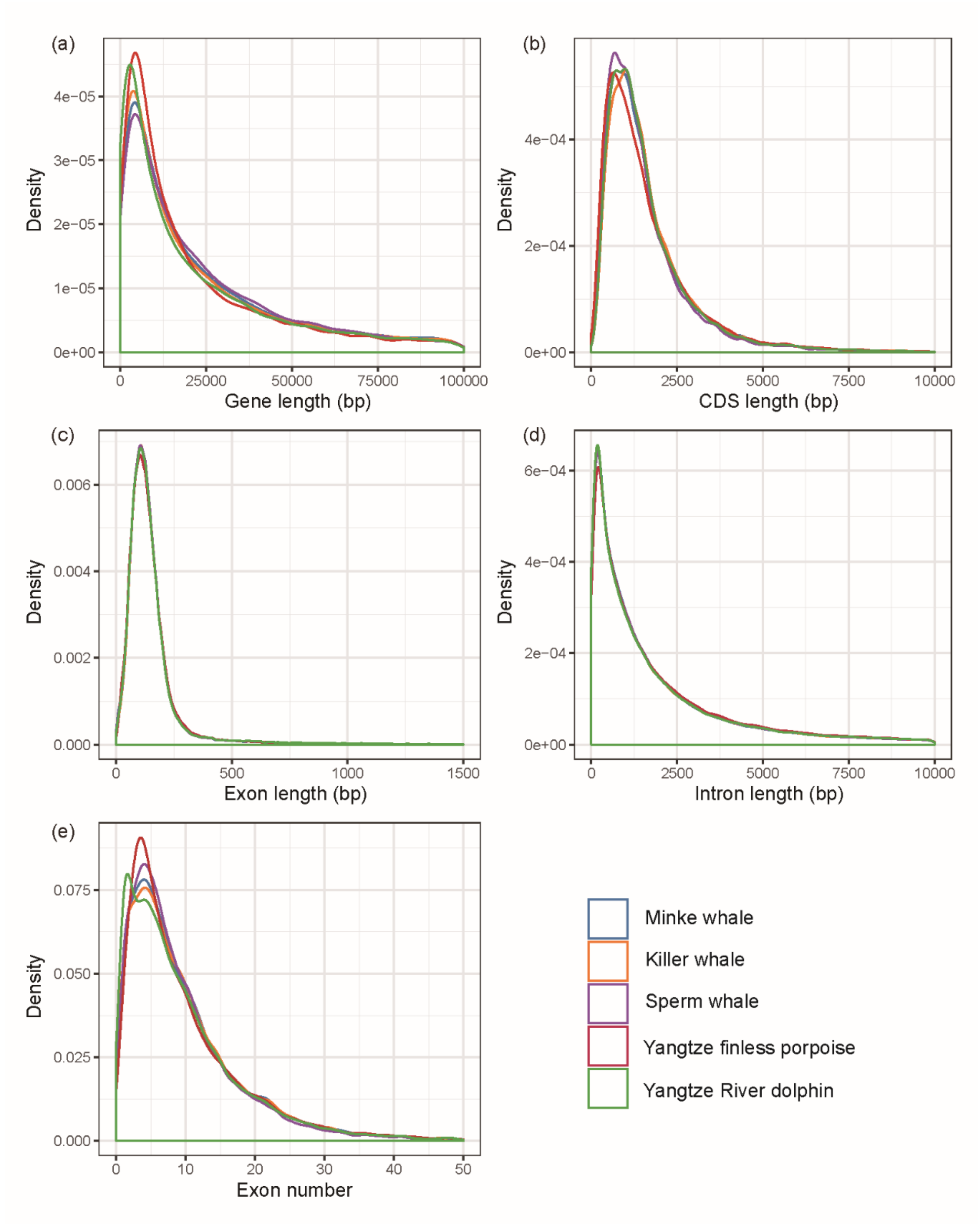

Figure S3. Comparison of gene structure characteristics of Yangtze finless porpoise and other cetaceans. The x-axis represents the length of corresponding genetic element of exon number and the y-axis represents gene density.

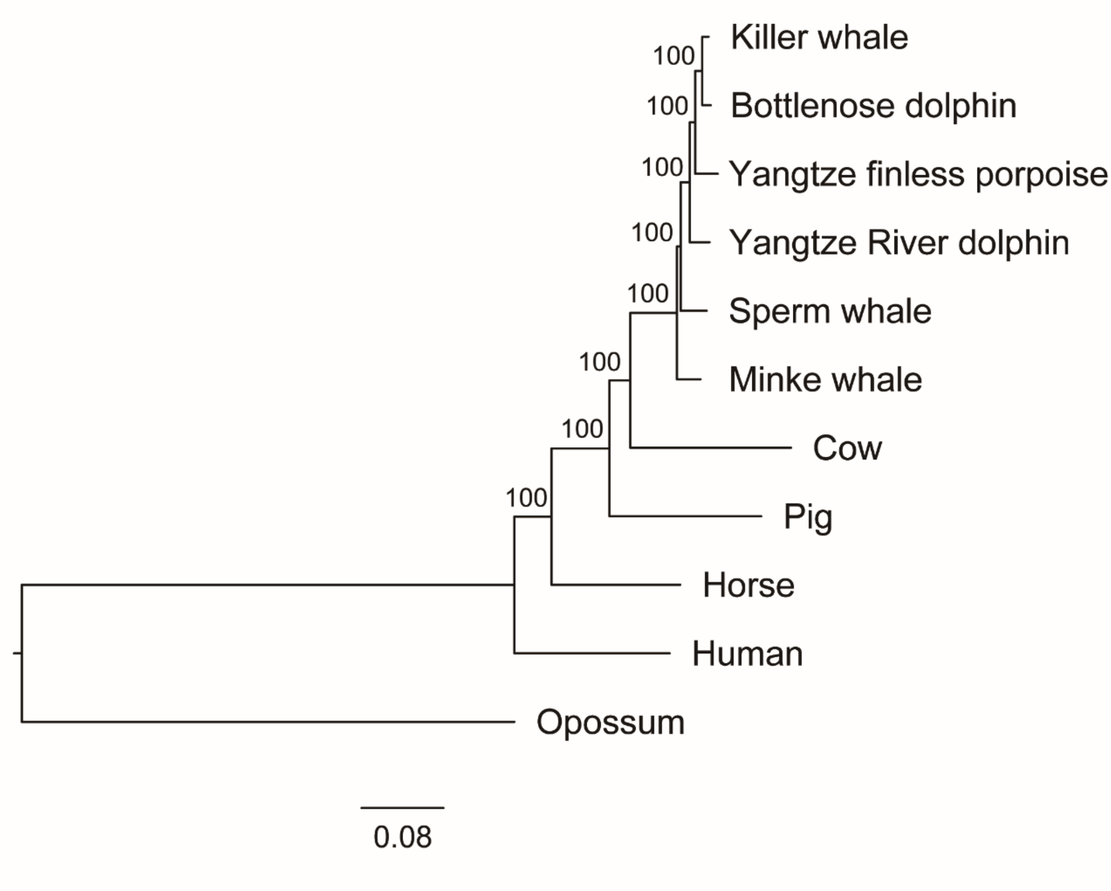

Figure S4. Phylogeny relationships between the Yangtze finless porpoise and other mammals reconstructed by RAxML with the GTR+G+I model.

Table S1. Summary of sequenced reads

	Raw Reads				Qualified Reads[1]				
Library Insert Size (bp)	Total Data (Gb)	Read Length (bp)	Sequence Coverage[2] (×)	Physical Coverage[2] (×)	Total Data (Gb)	Read Length (bp)	Sequence Coverage[2] (×)	Physical Coverage[2] (×)	SRA Number
289	58.94	150.00	23.67	22.80	57.84	149.75	23.23	22.41	SRR6923836
462	71.33	150.00	28.65	44.12	70.12	149.74	28.16	43.44	SRR6923837
624	67.47	150.00	27.10	56.36	63.90	149.67	25.66	53.50	SRR6923834
791	57.58	150.00	23.12	60.97	55.39	149.67	22.24	58.78	SRR6923835
4,000	108.73	150.00	43.67	582.22	70.74	150.00	28.41	378.80	SRR6923832
7,000	115.4	150.00	46.35	1,081.39	84.76	150.00	34.04	794.27	SRR6923833
11,000	107.37	150.00	43.12	1,581.08	79.78	150.00	32.04	1,174.81	SRR6923830
18,000	127.46	150.00	51.19	3,071.33	97.75	150.00	39.26	2,355.42	SRR6923831
**Total**	**714.28**	**-**	**286.87**	**6,500.27**	**580.28**	**-**	**233.04**	**4,881.43**	**-**

[1]Raw reads in mate-paired libraries were filtered to remove duplicates and reads with low quality and/or adapter contamination, raw reads in paired-end libraries were filtered in the same manner then subjected to k-mer-based correction.

[2]Coverage was calculated using an estimated Yangtze finless porpoise genome size of 2.5 Gb. Sequence coverage refers to the total length of generated reads, and physical coverage refers to the total cloned DNA used for the paired reads.

Table S2. 17-mer depth distribution.

K-mer value	K-mer_Num	Peak_Depth	Genome Size	Used Bases	Used Reads	Depth(x)
17	211,733,348,694	85	2,490,980,572	240,621,642,121	1,616,373,163	95.24

Table S3. Statistics for the final assemblies of the Yangtze finless porpoise genome

	Contig		Scaffold	
	**Size (bp)**	**Number**	**Size (bp)**	**Number**
**N90**	13,158	49,727	405,799	1,478
**N80**	21,552	36,306	692,029	1,042
**N70**	29,550	27,239	996,516	763
**N60**	37,733	20,364	1,337,316	562
**N50**	46,692	14,892	1,704,448	407
**Longest**	367,721		8,041,833	
**Total Size**	2,297,280,777		2,324,302,892	
**Average length**	21,911.42		95,897.00	

Table S4. Summary of BUSCO analysis of matches to the 4,104 mammalian BUSCOs

	Count	Ratio
**Complete BUSCOs**	3,838	93.60%
**Complete and single-copy BUSCOs**	3,807	92.80%
**Complete and duplicated BUSCOs**	31	0.80%
**Fragmented BUSCOs**	132	3.20%
**Missing BUSCOs**	165	4.10%

Table S5. Prediction of repetitive elements in the assembled Yangtze finless porpoise genome

Type	Repeat Size (bp)	% of genome
**TRF**	36,679,712	1.65
**RepeatMasker**	41,255,099	1.86
**RepeatProteinMask**	285,727,584	12.87
**De novo**	824,692,743	37.15
**Total**	887,629,810	39.98

Table S6. Summary statistics of interspersed repeat regions

Type	Repbase TEs		TE proteins		De novo		Combined TEs	
	Length (bp)	% in genome	Length (bp)	% in genome	Length (bp)	% in genome	Length (bp)	% in genome
**DNA**	13,030,245	0.59	5,742,922	0.26	58,599,942	2.64	70,751,802	3.19
**LINE**	14,122,460	0.64	274,740,639	12.38	551,867,994	24.86	607,756,082	27.38
**SINE**	5,488	0.00	0	0	106,612,802	4.80	106,617,381	4.80
**LTR**	2,395,432	0.11	5,257,279	0.24	90,096,068	4.06	94,925,729	4.28
**Other**	14,148,952	0.64	699	0.00	27,594,115	1.24	37,361,001	1.68
**Unknown**	362,336	0.02	0	0	3,922,485	0.18	4,284,821	0.19
**Total**	41,255,099	1.86	285,727,584	12.87	824,692,743	37.15	850,996,764	38.33

Table S7. Data on all species used during the genome analysis

Species	Latin name	Version	Source
**Cow**	Bos taurus	UMD_3.1.1	Ensembl
**Pig**	Sus scrofa	Sscrofa10.2	Ensembl
**Opossum**	Monodelphis domestica	MonDom5	Ensembl
**Horse**	Equus caballus	EquCab2	Ensembl
**Human**	Homo sapiens	GRCh38	Ensembl
**Yangtze River dolphin**	Lipotes vexillifer	Lipotes_vexillifer_v1	NCBI
**Bottlenose dolphin**	Tursiops truncatus	NIST Tur_tru v1	NCBI
**Killer whale**	Orcinus orca	Oorc_1.1	NCBI
**Common minke whale**	Balaenoptera acutorostrata	BalAcu1.0	NCBI
**Sperm whale**	Physeter catodon	Physeter_macrocephalus-2.0.2	NCBI

Table S8. Prediction of protein-coding genes in the Yangtze finless porpoise

Gene set		Total Genes Predicted	Average Gene Length(bp)	Average CDS Length(bp)	Average Exon per Gene	Average Exon Length(bp)	Average Intron Length(bp)
**De novo**	Augustus	37,303	28,264.61	921.47	6.25	147.40	5,738.43
	GlimmerHMM	85,860	6,800.71	489.92	3.44	142.38	2,728.15
	Geneid	134,560	9,110.22	526.35	4.11	128.03	2,876.60
**Homolog**	Cow	27695.00	29558.89	1287.05	6.87	187.29	8,717.24
	Killer whale	26942.00	32091.07	1356.77	7.37	184.21	7,706.16
	Sperm whale	26836.00	26501.65	1271.89	7.04	180.71	7,912.74
	Common minke whale	26332.00	30653.48	1318.68	7.35	179.52	7,995.26
	Yangtze River dolphin	26007.00	31493.15	1344.52	7.37	182.39	7,865.01
	Bottlenose dolphin	24622.00	31536.45	1266.24	6.79	186.47	8,077.46
**Final set**	-	18479	36929.68	1687.41	10.17	165.89	3,842.38

Table S9. Summary statistics of comparative gene structure

Gene set	Numbers	Average Gene Length (bp)	Average CDS Length (bp)	Average Exons per Gene	Average Exon Length (bp)	Average Intron Length (bp)
**Yangtze finless porpoise**	18,479	36,929.68	1,687.41	10.17	165.89	3,842.38
**Yangtze river dolphin**	18,877	45,085.80	1,697.09	9.92	180.00	4,613.82
**Killer whale**	18,129	51,256.47	1,749.02	10.30	169.76	4,949.68
**Sperm whale**	18,626	37,852.40	1,603.08	9.67	165.74	3,794.73
**Common minke whale**	18,400	50,781.71	1,697.13	10.28	165.10	4,702.13

Table S10. Summary of the predicted pseudogenes

Type	Numbers	% of genome
**Frame-shifted genes**	2373	93.79
**Prematurely terminated genes**	2131	84.23
**Frame-shifted and prematurely terminated genes**	1974	78.02
**Total**	2530	100

Table S11. Functional annotation of predicted genes in the Yangtze finless porpoise genome

	Database	Number	% of genome
**Total**		18,479	100
	InterPro	17,507	94.74
	GO	11,997	64.92
**Annotated**	Swiss-Prot	15,807	85.54
	TrEMBL	15,970	86.42
	KEGG	8,529	46.16
**Unannotated**		102	0.55

Table S12. Summary statistics of gene families in 11 species

Species	Total genes	Genes in families	Non-clustered genes	Families	Unique families	Genes per family	Maximum gene family size
**Cow**	19,981	18,197	1,784	17,157	114	1.06	15
**Pig**	22,410	18,891	3,519	17,512	179	1.08	21
**Human**	22,813	21,385	1,428	17,781	383	1.20	18
**Opossum**	21,313	14,262	7,051	12,248	479	1.16	27
**Horse**	20,431	17,884	2,547	16,700	192	1.07	18
**Yangtze finless porpoise**	18,479	14,072	4,407	13,911	44	1.01	5
**Yangtze River dolphin**	17,905	16,626	1,279	16,187	159	1.03	12
**Common minke whale**	17,525	16,340	1,185	16,071	25	1.02	8
**Killer whale**	17,283	16,658	625	16,493	5	1.01	4
**Sperm whale**	17,579	15,621	1,958	15,425	6	1.01	5
**Bottlenose dolphin**	16,531	13,400	3,131	13,337	6	1.00	7

Table S13. GO enrichment analysis of the unique gene families in the Yangtze finless porpoise lineage

GO	Type	Function	Adjust P-value
GO:0001518	Cellular component	voltage-gated sodium channel complex	0.012289
GO:0005248	Molecular function	voltage-gated sodium channel activity	0.012437
GO:0005272	Molecular function	sodium channel activity	0.016213
GO:0005787	Cellular component	signal peptidase complex	0.008365
GO:0015081	Molecular function	sodium ion transmembrane transporter activity	0.021554
GO:0034706	Cellular component	sodium channel complex	0.012289
GO:0071205	Biological process	protein localization to juxtaparanode region of axon	0.008439
GO:0099612	Biological process	protein localization to axon	0.008439

Table S14. GO enrichment analysis of the expanded gene families in the Yangtze finless porpoise lineage

GO	Type	Function	Adjust P-value
GO:0006325	biological process	chromatin organization	0.000391
GO:0007155	biological process	cell adhesion	0.002884
GO:0007156	biological process	homophilic cell adhesion via plasma membrane adhesion molecules	9.82E-09
GO:0016192	biological process	vesicle-mediated transport	0.012336
GO:0016337	biological process	single organismal cell-cell adhesion	4.41E-07
GO:0022610	biological process	biological adhesion	0.002884
GO:0051276	biological process	chromosome organization	0.005419
GO:0000786	cellular component	nucleosome	9.82E-09
GO:0005576	cellular component	extracellular region	0.049732
GO:0005622	cellular component	intracellular	0.000606
GO:0005886	cellular component	plasma membrane	8.76E-07
GO:0032993	cellular component	protein-DNA complex	1.21E-08
GO:0044427	cellular component	chromosomal part	1.74E-08
GO:0003779	molecular function	actin binding	0.002109
GO:0005488	molecular function	binding	0.010055
GO:0005509	molecular function	calcium ion binding	6.45E-07
GO:0005515	molecular function	protein binding	0.005586
GO:0046982	molecular function	protein heterodimerization activity	4.05E-05
GO:0046983	molecular function	protein dimerization activity	0.048739

Table S15. Candidate PSGs in the Yangtze finless porpoise lineage.

GeneID	Common name	Description
nas20645	NEXN	nexilin F-actin binding protein
nas02916	NA	NA
nas23431	XYLT2	xylosyltransferase 2
nas16008	STK11IP	serine/threonine kinase 11 interacting protein
nas05569	TNFRSF11B	TNF receptor superfamily member 11b
nas00047	NA	NA
nas11931	SUSD3	sushi domain containing 3
nas03498	PROKR1	prokineticin receptor 1
nas03109	PNLIP	pancreatic lipase
nas07571	CCDC40	coiled-coil domain containing 40
nas00173	LRRC3B	leucine rich repeat containing 3B
nas08339	CATSPER4	cation channel sperm associated 4
nas16933	KIF2C	kinesin family member 2C
nas11807	ANXA5	annexin A5
nas06894	MRPS16	mitochondrial ribosomal protein S16
nas06377	FANCL	Fanconi anemia complementation group L
nas12209	TAF1D	TATA-box binding protein associated factor, RNA polymerase I subunit D
nas01941	EFHB	EF-hand domain family member B
nas06520	ELANE	elastase, neutrophil expressed
nas12470	DDX43	DEAD-box helicase 43
nas00446	SLC35E4	solute carrier family 35 member E4
nas25099	VSX1	visual system homeobox 1
nas14990	EPB41L5	erythrocyte membrane protein band 4.1 like 5
nas17486	RASGEF1B	RasGEF domain family member 1B
nas12726	OGFR	opioid growth factor receptor
nas23659	DENND4C	DENN domain containing 4C
nas05407	ECSIT	ECSIT signalling integrator
nas23555	VSIG8	V-set and immunoglobulin domain containing 8
nas24488	EXOC2	exocyst complex component 2
nas06483	NA	NA
nas18892	STAG2	stromal antigen 2
nas04427	SLC9A8	solute carrier family 9 member A8
nas14634	KTN1	kinectin 1
nas11914	LRRCC1	leucine rich repeat and coiled-coil centrosomal protein 1
nas09087	SPINK5	serine peptidase inhibitor, Kazal type 5
nas21102	PLA2G4D	phospholipase A2 group IVD
nas02847	IL4	interleukin 4
nas10153	RARRES2	retinoic acid receptor responder 2
nas14533	RGCC	regulator of cell cycle
nas21249	NA	NA

nas21253	BCL7B	BCL tumor suppressor 7B
nas23250	CHMP4C	charged multivesicular body protein 4C
nas06877	KRT85	keratin 85
nas12651	GPR37	G protein-coupled receptor 37
nas02770	RAD17	RAD17 checkpoint clamp loader component
nas14796	ERAP2	endoplasmic reticulum aminopeptidase 2
nas21908	LPXN	leupaxin
nas17556	NA	NA
nas15084	EIF2B5	eukaryotic translation initiation factor 2B subunit epsilon
nas17645	NOC2L	NOC2 like nucleolar associated transcriptional repressor
nas17643	PLEKHN1	pleckstrin homology domain containing N1
nas08416	TEX9	testis expressed 9
nas00929	NA	NA
nas02338	FAM71B	family with sequence similarity 71 member B
nas23647	NA	NA
nas13737	SLC15A1	solute carrier family 15 member 1
nas09917	VPS16	VPS16, CORVET/HOPS core subunit
nas18950	PTPN14	protein tyrosine phosphatase, non-receptor type 14
nas18898	RHAG	Rh-associated glycoprotein
nas16263	CACNA1E	calcium voltage-gated channel subunit alpha1 E
nas23642	CRYL1	crystallin lambda 1
nas01898	RTBDN	retbindin
nas23373	GUCA2B	guanylate cyclase activator 2B
nas16726	SLC19A1	solute carrier family 19 member 1
nas07560	C1QTNF1	C1q and TNF related 1
nas14162	ZMYND8	zinc finger MYND-type containing 8
nas17483	BMP3	bone morphogenetic protein 3
nas13021	BLZF1	basic leucine zipper nuclear factor 1
nas14440	LIPN	lipase family member N
nas15025	BLCAP	bladder cancer associated protein
nas20965	GTSE1	G2 and S-phase expressed 1
nas00787	ARMCX5	armadillo repeat containing, X-linked 5
nas02736	VIP	vasoactive intestinal peptide
nas08359	NA	NA
nas20041	C1QL4	complement C1q like 4
nas16311	AKIP1	A-kinase interacting protein 1
nas24631	TBX21	T-box 21
nas09814	FAM214A	family with sequence similarity 214 member A
nas24863	CLRN3	clarin 3
nas10471	UBXN2B	UBX domain protein 2B
nas04036	KDELR2	KDEL endoplasmic reticulum protein retention receptor 2
nas14981	PHKA2	phosphorylase kinase regulatory subunit alpha 2
nas00035	ADGRG2	adhesion G protein-coupled receptor G2
nas08813	RAD18	RAD18, E3 ubiquitin protein ligase

nas22738	LTN1	listerin E3 ubiquitin protein ligase 1
nas13389	CRTC3	CREB regulated transcription coactivator 3
nas03431	TRIT1	tRNA isopentenyltransferase 1
nas12711	ATP5A1	ATP synthase, H+ transporting, mitochondrial F1 complex, alpha subunit 1, cardiac muscle
nas00078	TMEM214	transmembrane protein 214
nas02273	BTBD16	BTB domain containing 16
nas16279	PLAA	phospholipase A2 activating protein
nas06924	TBC1D31	TBC1 domain family member 31
nas23980	CFAP43	cilia and flagella associated protein 43
nas15028	SRC	SRC proto-oncogene, non-receptor tyrosine kinase
nas06412	FAN1	FANCD2 and FANCI associated nuclease 1
nas17320	CLEC7A	C-type lectin domain containing 7A
nas00840	SLCO2B1	solute carrier organic anion transporter family member 2B1
nas24601	GIP	gastric inhibitory polypeptide
nas05360	ARMC6	armadillo repeat containing 6
nas24309	FCRL5	Fc receptor like 5
nas13752	TIAM2	T-cell lymphoma invasion and metastasis 2
nas13755	NOX3	NADPH oxidase 3
nas06526	NFKBIZ	NFKB inhibitor zeta
nas01885	STX10	syntaxin 10
nas21060	NA	NA
nas24642	PSMB3	proteasome subunit beta 3
nas25017	CCDC175	coiled-coil domain containing 175
nas04180	SKAP2	src kinase associated phosphoprotein 2
nas13023	DSC2	desmocollin 2
nas00497	HEATR5B	HEAT repeat containing 5B
nas00495	EIF2AK2	eukaryotic translation initiation factor 2 alpha kinase 2
nas21622	HEMK1	HemK methyltransferase family member 1
nas17200	PHF1	PHD finger protein 1
nas24574	AARS2	alanyl-tRNA synthetase 2, mitochondrial
nas15423	WASF3	WAS protein family member 3
nas11540	CMPK2	cytidine/uridine monophosphate kinase 2
nas05284	BFSP1	beaded filament structural protein 1
nas07161	HMOX1	heme oxygenase 1
nas02501	SS18	SS18, nBAF chromatin remodeling complex subunit
nas04387	CCDC138	coiled-coil domain containing 138
nas03644	TTC29	tetratricopeptide repeat domain 29
nas05211	EFCAB7	EF-hand calcium binding domain 7
nas09873	THEMIS2	thymocyte selection associated family member 2
nas02725	NPY	neuropeptide Y
nas11758	RIPPLY1	ripply transcriptional repressor 1
nas08797	IL17RC	interleukin 17 receptor C

nas08790	BRK1	BRICK1, SCAR/WAVE actin nucleating complex subunit
nas05020	NA	NA
nas22824	MRAP	melanocortin 2 receptor accessory protein
nas11266	LUZP1	leucine zipper protein 1
nas17864	NA	NA
nas17760	CASP10	caspase 10
nas14017	DENND1C	DENN domain containing 1C
nas22183	OPN4	opsin 4
nas13387	FURIN	furin, paired basic amino acid cleaving enzyme
nas21340	COX4I2	cytochrome c oxidase subunit 4I2
nas13635	NECTIN2	nectin cell adhesion molecule 2
nas05854	C16orf46	chromosome 16 open reading frame 46
nas23355	FAM183A	family with sequence similarity 183 member A
nas14724	GABRA3	gamma-aminobutyric acid type A receptor alpha3 subunit
nas12913	COIL	coilin
nas22178	SNCG	synuclein gamma
nas04138	BRCC3	BRCA1/BRCA2-containing complex subunit 3
nas17850	RPL18	ribosomal protein L18
nas13823	IKZF3	IKAROS family zinc finger 3
nas19386	POU2F2	POU class 2 homeobox 2
nas08114	OS9	OS9, endoplasmic reticulum lectin
nas21262	CLDN3	claudin 3
nas24907	CFAP20	cilia and flagella associated protein 20
nas24604	CALCOCO2	calcium binding and coiled-coil domain 2
nas22293	PTPRC	protein tyrosine phosphatase, receptor type C
nas11780	ZBTB37	zinc finger and BTB domain containing 37
nas18336	SNIP1	Smad nuclear interacting protein 1
nas15225	TIFA	TRAF interacting protein with forkhead associated domain
nas23493	ALOX12	arachidonate 12-lipoxygenase, 12S type
nas18773	PHACTR2	phosphatase and actin regulator 2
nas07059	SOX13	SRY-box 13
nas22511	TSGA10IP	testis specific 10 interacting protein
nas12563	NA	NA
nas10397	SLC6A13	solute carrier family 6 member 13
nas22457	MAP4K2	mitogen-activated protein kinase kinase kinase kinase 2
nas20120	C7orf61	chromosome 7 open reading frame 61
nas22459	CDC42BPG	CDC42 binding protein kinase gamma
nas09242	C6orf163	chromosome 6 open reading frame 163
nas10521	SFTPB	surfactant protein B
nas06033	COA1	cytochrome c oxidase assembly factor 1 homolog
nas21046	TOMM40L	translocase of outer mitochondrial membrane 40 like
nas00137	PTGFR	prostaglandin F receptor
nas14104	TRIM21	tripartite motif containing 21

nas12278	HPS4	HPS4, biogenesis of lysosomal organelles complex 3 subunit 2
nas00327	FANCE	Fanconi anemia complementation group E
nas21158	TCL1A	T-cell leukemia/lymphoma 1A
nas05392	SPC24	SPC24, NDC80 kinetochore complex component
nas05673	ZGLP1	zinc finger, GATA-like protein 1
nas24115	GTF3C1	general transcription factor IIIC subunit 1
nas07996	SORT1	sortilin 1
nas05172	SLC34A1	solute carrier family 34 member 1
nas23488	ASGR2	asialoglycoprotein receptor 2
nas00629	GDF11	growth differentiation factor 11
nas20894	ANGPTL4	angiopoietin like 4
nas16473	UTP6	UTP6, small subunit processome component
nas15045	NECAB3	N-terminal EF-hand calcium binding protein 3
nas07102	MATN1	matrilin 1, cartilage matrix protein
nas00433	EIF4ENIF1	eukaryotic translation initiation factor 4E nuclear import factor 1
nas13916	TMEM107	transmembrane protein 107
nas14660	NFIB	nuclear factor I B
nas14079	FOLR1	folate receptor 1
nas15332	ZYX	zyxin
nas06625	PRDX3	peroxiredoxin 3
nas12695	CHRNG	cholinergic receptor nicotinic gamma subunit
nas05791	ZUFSP	zinc finger with UFM1 specific peptidase domain
nas02353	MTG2	mitochondrial ribosome associated GTPase 2
nas20140	ACHE	acetylcholinesterase (Cartwright blood group)
nas05975	TSPAN6	tetraspanin 6
nas07005	TMEM175	transmembrane protein 175
nas08391	FIGLA	folliculogenesis specific bHLH transcription factor
nas16061	BSG	basigin (Ok blood group)
nas16675	RSPH3	radial spoke head 3 homolog
nas04884	SLC9A6	solute carrier family 9 member A6
nas13718	TMEM82	transmembrane protein 82
nas19561	MAN2C1	mannosidase alpha class 2C member 1
nas03250	NPPA	natriuretic peptide A
nas16171	APOA1	apolipoprotein A1
nas01817	ATP6V1H	ATPase H+ transporting V1 subunit H
nas10496	C11orf70	chromosome 11 open reading frame 70
nas00941	TMEM100	transmembrane protein 100
nas02506	AQP4	aquaporin 4
nas02782	PTCD2	pentatricopeptide repeat domain 2
nas19809	PIF1	PIF1 5'-to-3' DNA helicase
nas23909	BLOC1S2	biogenesis of lysosomal organelles complex 1 subunit 2
nas17899	DHRS11	dehydrogenase/reductase 11

nas12814	PLEKHS1	pleckstrin homology domain containing S1
nas16651	PACRG	parkin coregulated
nas10251	HR	HR, lysine demethylase and nuclear receptor corepressor
nas23761	MED31	mediator complex subunit 31
nas22303	MLANA	melan-A
nas14420	C1orf116	chromosome 1 open reading frame 116
nas02169	IRF2BPL	interferon regulatory factor 2 binding protein like
nas24032	LRP3	LDL receptor related protein 3
nas19115	GNA11	G protein subunit alpha 11
nas20985	HDAC10	histone deacetylase 10
nas15372	LEPROTL1	leptin receptor overlapping transcript like 1
nas17011	PTGDR2	prostaglandin D2 receptor 2
nas01425	10-Mar	membrane associated ring-CH-type finger 10
nas12118	C2CD2	C2 calcium dependent domain containing 2
nas24986	SRD5A1	steroid 5 alpha-reductase 1
nas11662	SEMA4F	ssemaphorin 4F
nas14131	TAF7L	TATA-box binding protein associated factor 7 like
nas07821	SHANK1	SH3 and multiple ankyrin repeat domains 1
nas12224	SETDB2	SET domain bifurcated 2
nas03421	ZFP69	ZFP69 zinc finger protein
nas22286	NEK6	NIMA related kinase 6
nas16761	KRT78	keratin 78
nas09226	CTH	cystathionine gamma-lyase
nas10143	GIMAP4	GTPase, IMAP family member 4
nas02963	CSN2	casein beta
nas09542	SOAT1	sterol O-acyltransferase 1
nas19918	CABP4	calcium binding protein 4
nas15431	ADAMTS18	ADAM metallopeptidase with thrombospondin type 1 motif 18
nas05505	FAM151A	family with sequence similarity 151 member A
nas12372	DNA2	DNA replication helicase/nuclease 2
nas22425	RTN3	reticulon 3
nas02123	PARP9	poly(ADP-ribose) polymerase family member 9
nas18821	EFR3A	EFR3 homolog A
nas02489	USP26	ubiquitin specific peptidase 26
nas08655	LCLAT1	lysocardiolipin acyltransferase 1
nas04869	ZCCHC9	zinc finger CCHC-type containing 9
nas06547	C8orf33	chromosome 8 open reading frame 33
nas13946	PRR19	proline rich 19
nas18176	PSCA	prostate stem cell antigen
nas04830	CEP85L	centrosomal protein 85 like
nas15940	C1D	C1D nuclear receptor corepressor
nas23802	ZBTB4	zinc finger and BTB domain containing 4
nas05300	SLC24A3	solute carrier family 24 member 3

nas22601	C9orf84	chromosome 9 open reading frame 84
nas03486	CCDC158	coiled-coil domain containing 158
nas19233	CEL	carboxyl ester lipase
nas14309	MSANTD1	Myb/SANT DNA binding domain containing 1
nas07598	IL21	interleukin 21
nas09693	TOR1AIP2	torsin 1A interacting protein 2
nas19633	ISG20L2	interferon stimulated exonuclease gene 20 like 2
nas15507	EPC2	enhancer of polycomb homolog 2
nas04245	CD8A	CD8a molecule
nas15934	FYN	FYN proto-oncogene, Src family tyrosine kinase
nas06753	RPS19BP1	ribosomal protein S19 binding protein 1
nas02468	IMPACT	impact RWD domain protein
nas02841	GDF9	growth differentiation factor 9
nas00012	RB1CC1	RB1 inducible coiled-coil 1
nas13960	IRGQ	immunity related GTPase Q
nas01117	SOWAHB	sosondowah ankyrin repeat domain family member B
nas00300	SYCN	syncollin
nas06184	LAMTOR3	late endosomal/lysosomal adaptor, MAPK and MTOR activator 3
nas00203	TM4SF20	transmembrane 4 L six family member 20
nas22837	IFNAR1	interferon alpha and beta receptor subunit 1
nas13299	HVCN1	hydrogen voltage gated channel 1
nas22007	MSX2	msh homeobox 2
nas17247	TRIM45	tripartite motif containing 45
nas20838	TBXAS1	thromboxane A synthase 1
nas19492	ICK	intestinal cell kinase
nas20939	SAMM50	SAMM50 sorting and assembly machinery component
nas05944	PSMG1	proteasome assembly chaperone 1
nas04365	ZNF830	zinc finger protein 830
nas08305	SYTL1	synaptotagmin like 1
nas24189	SETD1A	SET domain containing 1A
nas06006	CXXC1	CXXC finger protein 1
nas05516	TMEM61	transmembrane protein 61
nas16426	CYTIP	cytohesin 1 interacting protein
nas13193	PLSCR4	phospholipid scramblase 4
nas03791	TVP23A	trans-golgi network vesicle protein 23 homolog A
nas20412	CTCFL	CCCTC-binding factor like
nas02042	LRSAM1	leucine rich repeat and sterile alpha motif containing 1
nas13726	CTRC	chymotrypsin C
nas20320	HGFAC	HGF activator
nas22035	APOD	apolipoprotein D
nas01537	SAP30BP	SAP30 binding protein
nas20730	COX4I1	cytochrome c oxidase subunit 4I1
nas24417	NCAPD2	non-SMC condensin I complex subunit D2

nas24415	CHD4	chromodomain helicase DNA binding protein 4
nas16067	SHC2	SHC adaptor protein 2
nas16466	RHOT1	ras homolog family member T1
nas21769	NA	NA
nas16305	RIC3	RIC3 acetylcholine receptor chaperone
nas24229	RITA1	RBPJ interacting and tubulin associated 1
nas13836	CASC3	cancer susceptibility 3
nas17630	C1QTNF12	C1q and TNF related 12
nas17624	INTS11	integrator complex subunit 11
nas18562	CFTR	cystic fibrosis transmembrane conductance regulator
nas07183	TPRA1	transmembrane protein adipocyte associated 1
nas24879	KAZN	kazrin, periplakin interacting protein
nas19370	TEX14	testis expressed 14, intercellular bridge forming factor
nas05071	TEPSIN	TEPSIN, adaptor related protein complex 4 accessory protein
nas15426	CAGE1	cancer antigen 1
nas08634	FADS1	fatty acid desaturase 1

Table S16. The classification of the candidate PSGs

Gene ID	PATHER ID	description
nas20645	PTHR10489	cell adhesion molecule
nas02916	PTHR15004	uncharacterized
nas23431	PTHR19297	glycosyltransferase 14 family member
nas16008	PTHR15454	nischarin related
nas05569	PTHR23097	tumor necrosis factor receptor superfamily member
nas00047	PTHR23053	dlec1 deleted in lung and esophageal cancer 1
nas11931	PTHR19325	complement component-related sushi domain-containing
nas03498	PTHR24242	g-protein coupled receptor
nas03109	PTHR11610	lipase
nas07571	PTHR22762	alpha-glucosidase
nas00173	PTHR24369	family not named
nas08339	PTHR10037	voltage-gated cation channel calcium and sodium
nas16933	PTHR24115	family not named
nas11807	PTHR10502	annexin
nas06894	PTHR12919	30s ribosomal protein s16
nas06377	PTHR13206	ubiquitin ligase protein phf9 fanconi anemia group l protein
nas12209	PTHR14562	family not named
nas01941	PTHR12086	ef-hand domain c-terminal containing protein
nas06520	PTHR24257	chymotrypsin-like elastase family member
nas12470	PTHR24031	rna helicase
nas00446	PTHR11132	solute carrier family 35
nas25099	PTHR24323	family not named
nas14990	PTHR23280	4.1 g protein
nas17486	PTHR23113	guanine nucleotide exchange factor
nas12726	PTHR14015	opioid growth factor receptor ogfr zeta-type opioid receptor
nas23659	PTHR12296	c-myc promoter binding protein
nas05407	PTHR13113	ecsit evolutionarily conserved signaling intermediate in toll pathways
nas23555	PTHR12231	ctx-related type i transmembrane protein
nas24488	PTHR13043	exocyst complex component sec5
nas06483	PTHR24375	NA
nas18892	PTHR11199	stromal antigen
nas04427	PTHR10110	sodium/hydrogen exchanger
nas14634	PTHR18864	kinectin
nas11914	PTHR24365	toll-like receptor
nas09087	PTHR10913	follistatin-related
nas21102	PTHR10728	cytosolic phospholipase a2
nas02847	PTHR10078	interleukin-1 family member
nas10153	PTHR15106	family not named

nas14533	PTHR32193	family not named
nas21249	PTHR24103	trim/rbcc ring finger, b-box and coiled coil domains-containing
nas21253	PTHR12767	bcl7 related
nas23250	PTHR22761	snf7 - related
nas06877	PTHR23239	intermediate filament
nas12651	PTHR24243	g-protein coupled receptor
nas02770	PTHR12172	cell cycle checkpoint protein rad17
nas14796	PTHR11533	protease m1 zinc metalloprotease
nas21908	PTHR24216	family not named
nas17556	PTHR11955	fatty acid binding protein
nas15084	PTHR22572	sugar-1-phosphate guanyl transferase
nas17645	PTHR12687	nucleolar complex 2 and rad4-related
nas17643	PTHR22826	rho guanine exchange factor-related
nas08416	PTHR23313	tsec1-related
nas00929	PTHR12670	ceramidase
nas02338	PTHR22574	uncharacterized
nas23647	PTHR11697	general transcription factor 2-related zinc finger protein
nas13737	PTHR11654	oligopeptide transporter-related
nas09917	PTHR12811	vacuolar protein sorting vps16
nas18950	PTHR19134	protein-tyrosine phosphatase
nas18898	PTHR11730	ammonium transporter
nas16263	PTHR10037	voltage-gated cation channel calcium and sodium
nas23642	PTHR43492	family not named
nas01898	PTHR10517	folate receptor
nas23373	PTHR11318	guanylin family member
nas16726	PTHR10686	folate transporter
nas07560	PTHR22923	cerebellin-related
nas14162	PTHR24102	phd finger protein
nas17483	PTHR11848	tgf-beta family
nas13021	PTHR13066	basic leucine zipper nuclear factor 1 blzf1 protein
nas14440	PTHR11005	lysosomal acid lipase-related
nas15025	PTHR13259	bladder cancer 10 kd protein homolog
nas20965	PTHR21584	differential display and activated by p53 dda3 /g2 s phase expressed 1
nas00787	PTHR15712	armadillo repeat containing protein
nas02736	PTHR11213	glucagon-family neuropeptide
nas08359	PTHR11999	group ii pyridoxal-5-phosphate decarboxylase
nas20041	PTHR22923	cerebellin-related
nas16311	PTHR14330	family not named
nas24631	PTHR11267	t-box protein-related
nas09814	PTHR13199	family not named
nas24863	PTHR31548	family not named
nas10471	PTHR23333	ubx domain containing protein

nas04036	PTHR10585	er lumen protein retaining receptor
nas14981	PTHR10749	phosphorylase b kinase regulatory subunit
nas00035	PTHR12011	g-protein coupled receptor
nas08813	PTHR14134	e3 ubiquitin-protein ligase rad18
nas22738	PTHR12389	zinc finger protein 294
nas13389	PTHR13589	transducer of regulated creb protein
nas03431	PTHR11088	trna delta 2 -isopentenylpyrophosphate transferase-related
nas12711	PTHR43089	family not named
nas00078	PTHR13448	uncharacterized
nas02273	PTHR23231	family not named
nas16279	PTHR19849	phospholipase a-2-activating protein
nas06924	PTHR19853	wd repeat containing protein 3 wdr3
nas23980	PTHR14885	uncharacterized
nas15028	PTHR24418	tyrosine-protein kinase
nas06412	PTHR15749	family not named
nas17320	PTHR22800	c-type lectin proteins
nas00840	PTHR11388	organic anion transporter
nas24601	PTHR15211	glucose-dependent insulinotropic polypeptide
nas05360	PTHR22895	uncharacterized
nas24309	PTHR11481	immunoglobulin fc receptor
nas13752	PTHR22826	rho guanine exchange factor-related
nas13755	PTHR11972	nadph oxidase
nas06526	PTHR24124	family not named
nas01885	PTHR19957	syntaxin
nas21060	PTHR43205	family not named
nas24642	PTHR11599	proteasome subunit alpha/beta
nas25017	PTHR35347	family not named
nas04180	PTHR15129	src-associated adaptor protein
nas13023	PTHR24025	family not named
nas00497	PTHR21663	hypothetical heat domain-containing
nas00495	PTHR11042	eukaryotic translation initiation factor 2-alpha kinase eif2-alpha kinase -related
nas21622	PTHR18895	methyltransferase
nas17200	PTHR12628	polycomb-like transcription factor
nas24574	PTHR11777	alanyl-trna synthetase
nas15423	PTHR12902	wasp-1
nas11540	PTHR10344	thymidylate kinase
nas05284	PTHR14069	filensin
nas07161	PTHR10720	heme oxygenase
nas02501	PTHR23107	synovial sarcoma associated ss18 protein
nas04387	PTHR34523	family not named
nas03644	PTHR10098	rapsyn-related
nas05211	PTHR23050	calcium binding protein
nas09873	PTHR15215	family not named

nas02725	PTHR10533	neuropeptide y/pancreatic hormone/peptide yy
nas11758	PTHR16770	family not named
nas08797	PTHR15583	interleukin-17 receptor
nas08790	PTHR33668	family not named
nas05020	PTHR11595	elongation factor 1-beta
nas22824	PTHR28675	family not named
nas11266	PTHR23166	filamin/gpbp-interacting protein
nas17864	PTHR21694	uncharacterized
nas17760	PTHR10454	caspase
nas14017	PTHR13196	denn domain-containing
nas22183	PTHR24240	opsin
nas13387	PTHR42884	family not named
nas21340	PTHR10707	cytochrome c oxidase subunit iv
nas13635	PTHR10802	mitochondrial import receptor subunit tom40
nas05854	PTHR36869	family not named
nas23355	PTHR33865	family not named
nas14724	PTHR18945	neurotransmitter gated ion channel
nas12913	PTHR15197	coilin p80
nas22178	PTHR13820	synuclein
nas04138	PTHR10410	eukaryotic translation initiation factor 3 -related
nas17850	PTHR10934	60s ribosomal protein l18
nas13823	PTHR24404	zinc finger protein
nas19386	PTHR11636	pou domain
nas08114	PTHR15414	os-9-related
nas21262	PTHR12002	claudin
nas24907	PTHR12458	orf protein
nas24604	PTHR31915	family not named
nas22293	PTHR19134	protein-tyrosine phosphatase
nas11780	PTHR24375	NA
nas18336	PTHR23308	nuclear inhibitor of protein phosphatase-1
nas15225	PTHR31266	family not named
nas23493	PTHR11771	lipoxygenase
nas18773	PTHR12751	phosphatase and actin regulator phactr
nas07059	PTHR10270	sox transcription factor
nas22511	PTHR21501	uncharacterized
nas12563	PTHR36866	family not named
nas10397	PTHR11616	sodium/chloride dependent transporter
nas22457	PTHR24361	mitogen-activated kinase
nas20120	PTHR39221	family not named
nas22459	PTHR22988	myotonic dystrophy s/t kinase-related
nas09242	PTHR34645	family not named
nas10521	PTHR11480	saposin-related
nas06033	PTHR10768	60s ribosomal protein l37
nas21046	PTHR10802	mitochondrial import receptor subunit tom40

nas00137	PTHR11866	prostaglandin receptor
nas14104	PTHR24103	trim/rbcc ring finger, b-box and coiled coil domains-containing
nas12278	PTHR14407	hermansky-pudlak syndrome 4 protein light-ear protein-related
nas00327	PTHR32094	family not named
nas21158	PTHR14060	family not named
nas05392	PTHR22142	uncharacterized
nas05673	NA	NA
nas24115	PTHR15180	general transcription factor 3c polypeptide 1
nas07996	PTHR12106	sortilin related
nas05172	PTHR10010	solute carrier family 34 sodium phosphate member 2-related
nas23488	PTHR22802	c-type lectin superfamily member
nas00629	PTHR11848	tgf-beta family
nas20894	PTHR19143	fibrinogen/tenascin/angiopoeitin
nas16473	PTHR23271	hepatocellular carcinoma-associated antigen 66
nas15045	PTHR12178	family not named
nas07102	PTHR11132	solute carrier family 35
nas00433	PTHR12269	eukaryotic translation initiation factor 4e transporter
nas13916	PTHR34341	family not named
nas14660	PTHR11492	nuclear factor i
nas14079	PTHR10517	folate receptor
nas15332	PTHR24212	family not named
nas06625	PTHR10681	thioredoxin peroxidase
nas12695	PTHR18945	neurotransmitter gated ion channel
nas05791	PTHR24375	NA
nas02353	PTHR11702	developmentally regulated gtp-binding protein-related
nas20140	PTHR11559	carboxylesterase
nas05975	PTHR19282	tetraspanin
nas07005	PTHR31462	family not named
nas08391	PTHR23349	basic helix-loop-helix transcription factor, twist
nas16061	PTHR10075	basigin related
nas16675	PTHR21648	flagellar radial spoke protein 3
nas04884	PTHR10110	sodium/hydrogen exchanger
nas13718	PTHR35257	family not named
nas19561	PTHR11607	alpha-mannosidase
nas03250	PTHR14066	atrial natriuretic factor precursor
nas16171	PTHR18976	apolipoprotein
nas01817	PTHR10698	v-type proton atpase subunit h
nas10496	PTHR31078	family not named
nas00941	PTHR16100	family not named
nas02506	PTHR19139	aquaporin transporter
nas02782	PTHR14700	family not named

nas19809	PTHR23274	dna helicase-related
nas23909	PTHR10668	phytoene dehydrogenase
nas17899	PTHR43115	family not named
nas12814	PTHR12156	grb2-associated binder, gab
nas16651	PTHR21207	parkin coregulated gene protein park2 coregulated
nas10251	PTHR12549	jmjc domain-containing histone demethylation protein
nas23761	PTHR13186	mediator of rna polymerase ii transcription subunit soh1
nas22303	PTHR15305	family not named
nas14420	PTHR21555	family not named
nas02169	PTHR10816	myelin transcription factor 1-related
nas24032	PTHR10529	low-density lipoprotein receptor-related
nas19115	PTHR10218	gtp-binding protein alpha subunit
nas20985	PTHR10625	histone deacetylase
nas15372	PTHR12050	leptin receptor-related
nas17011	PTHR24229	neuropeptides receptor
nas01425	PTHR14471	e3 ubiquitin-protein ligase march7, 10
nas12118	PTHR21119	uncharacterized
nas24986	PTHR10556	3-oxo-5-alpha-steroid 4-dehydrogenase
nas11662	PTHR11036	semaphorin
nas14131	PTHR12228	transcription initiation factor tfiid 55 kd subunit-related
nas07821	PTHR24135	family not named
nas12224	PTHR22884	set domain proteins
nas03421	PTHR24381	family not named
nas22286	PTHR43289	family not named
nas16761	PTHR23239	intermediate filament
nas09226	PTHR11808	trans-sulfuration enzyme family member
nas10143	PTHR10903	gtpase, imap family member-related
nas02963	PTHR11500	beta casein
nas09542	PTHR10408	sterol o-acyltransferase
nas19918	PTHR23050	calcium binding protein
nas15431	PTHR13723	adamts a disintegrin and metalloprotease with thrombospondin motifs protease
nas05505	PTHR21184	family not named
nas12372	PTHR10887	dna2/nam7 helicase family
nas22425	PTHR10994	reticulon
nas02123	PTHR14453	parp/zinc finger ccch type domain containing protein
nas18821	PTHR12444	uncharacterized
nas02489	PTHR24006	family not named
nas08655	PTHR10983	1-acylglycerol-3-phosphate acyltransferase-related
nas04869	PTHR23002	zinc finger cchc domain containing protein
nas06547	PTHR13602	uncharacterized
nas13946	PTHR37346	family not named
nas18176	PTHR16983	family not named
nas04830	PTHR31075	family not named

nas15940	PTHR15341	sun-cor steroid hormone receptor co-repressor
nas23802	PTHR19303	transposon
nas05300	PTHR10846	sodium/potassium/calcium exchanger
nas22601	PTHR35668	family not named
nas03486	PTHR13140	myosin
nas19233	PTHR11559	carboxylesterase
nas14309	PTHR22666	uncharacterized
nas07598	PTHR14356	family not named
nas09693	PTHR18843	torsin-1a-interacting protein
nas19633	PTHR12801	exonuclease
nas15507	PTHR14898	enhancer of polycomb
nas04245	PTHR10441	cd8 alpha chain
nas15934	PTHR24418	tyrosine-protein kinase
nas06753	PTHR31454	family not named
nas02468	PTHR16301	impact-related
nas02841	PTHR11848	tgf-beta family
nas00012	PTHR13222	cgthba protein -14 gene protein
nas13960	PTHR19364	family not named
nas01117	PTHR14491	uncharacterized
nas00300	PTHR17503	family not named
nas06184	PTHR13378	regulator complex protein lamtor3
nas00203	PTHR14198	family not named
nas22837	PTHR20859	interferon/interleukin receptor
nas13299	PTHR12305	phosphatase with homology to tensin
nas22007	PTHR24338	family not named
nas17247	PTHR24103	trim/rbcc ring finger, b-box and coiled coil domains-containing
nas20838	PTHR24301	family not named
nas19492	PTHR24055	mitogen-activated protein kinase
nas20939	PTHR12815	sorting and assembly machinery sam50 protein
nas05944	PTHR15069	family not named
nas04365	PTHR13278	uncharacterized
nas08305	PTHR10024	synaptotagmin
nas24189	PTHR22884	set domain proteins
nas06006	PTHR12321	cpg binding protein
nas05516	PTHR37151	family not named
nas16426	PTHR15963	general receptor for phosphoinositides 1-associated scaffold protein-related
nas13193	PTHR23248	phospholipid scramblase-related
nas03791	PTHR13019	uncharacterized
nas20412	PTHR24375	NA
nas02042	PTHR23155	leucine-rich repeat-containing protein
nas13726	PTHR24250	family not named
nas20320	PTHR24256	transmembrane protease, serine

nas22035	PTHR12398	protein phosphatase inhibitor
nas01537	PTHR13464	transcriptional regulator protein hcngp
nas20730	PTHR10707	cytochrome c oxidase subunit iv
nas24417	PTHR14222	condensin
nas24415	PTHR10799	swi/snf-related matrix-associated actin-dependent regulator of chromatin subfamily-related
nas16067	PTHR10337	shc transforming protein
nas16466	PTHR24072	rho family gtpase
nas21769	PTHR22765	ring finger and protease associated domain-containing
nas16305	PTHR21723	resistance to inhibitors of cholinesterase protein 3 ric3
nas24229	PTHR34917	family not named
nas13836	PTHR13434	family not named
nas17630	PTHR24019	family not named
nas17624	PTHR11203	cleavage and polyadenylation specificity factor
nas18562	PTHR24223	family not named
nas07183	PTHR15876	transmembrane protein adipocyte-associated 1
nas24879	PTHR12776	kazrin-related
nas19370	PTHR23060	testis expressed gene 14
nas05071	PTHR21514	uncharacterized
nas15426	PTHR36864	family not named
nas08634	PTHR19353	fatty acid desaturase 2

Table S16. GO categories showing accelerated evolutionary rates in the Yangtze finless porpoise lineage and the other cetaceans

GO	Type	Function	Adjust P-value
GO:0007601	biological process	visual perception	0.024435712
GO:0016192	biological process	vesicle-mediated transport	5.55E-11
GO:0006511	biological process	ubiquitin-dependent protein catabolic process	0.020800796
GO:0006810	biological process	transport	8.07E-34
GO:0055085	biological process	transmembrane transport	2.42E-08
GO:0044281	biological process	small molecule metabolic process	1.16E-13
GO:0007165	biological process	signal transduction	5.87E-31
GO:0032774	biological process	RNA biosynthetic process	0.018415765
GO:0009259	biological process	ribonucleotide metabolic process	8.91E-05
GO:0009611	biological process	response to wounding	2.04E-05
GO:0050896	biological process	response to stimulus	6.53E-36
GO:0009605	biological process	response to external stimulus	0.030767796
GO:0042221	biological process	response to chemical	6.26E-15
GO:0022414	biological process	reproductive process	3.27E-05
GO:0006357	biological process	regulation of transcription from RNA polymerase II promoter	5.30E-11
GO:0080134	biological process	regulation of response to stress	2.07E-11
GO:0048583	biological process	regulation of response to stimulus	3.09E-16
GO:0043067	biological process	regulation of programmed cell death	0.001688394
GO:0051239	biological process	regulation of multicellular organismal process	0.000215502
GO:0002682	biological process	regulation of immune system process	0.002036577
GO:0050776	biological process	regulation of immune response	0.009061234
GO:0050794	biological process	regulation of cellular process	5.53E-70
GO:0051128	biological process	regulation of cellular component organization	1.70E-12
GO:0031329	biological process	regulation of cellular catabolic process	4.00E-10
GO:0010941	biological process	regulation of cell death	0.001688394
GO:0009894	biological process	regulation of catabolic process	3.07E-11
GO:0065008	biological process	regulation of biological quality	4.32E-05
GO:0050789	biological process	regulation of biological process	2.42E-68
GO:0042981	biological process	regulation of apoptotic process	0.001688394
GO:0072521	biological process	purine-containing compound metabolic process	9.93E-07
GO:0072522	biological process	purine-containing compound biosynthetic process	2.35E-06
GO:0006163	biological process	purine nucleotide metabolic process	1.73E-05
GO:0006164	biological process	purine nucleotide biosynthetic process	2.18E-06
GO:0006508	biological process	proteolysis	6.73E-06
GO:0016567	biological process	protein ubiquitination	1.46E-07
GO:0015031	biological process	protein transport	1.23E-07
GO:0006468	biological process	protein phosphorylation	1.71E-09
GO:0036211	biological process	protein modification process	8.53E-17

GO:0070646	biological process	protein modification by small protein removal	0.010334405
GO:0019538	biological process	protein metabolic process	4.64E-23
GO:0016579	biological process	protein deubiquitination	0.010334405
GO:0012501	biological process	programmed cell death	0.007746723
GO:0044238	biological process	primary metabolic process	4.41E-48
GO:0006813	biological process	potassium ion transport	0.007305762
GO:0048584	biological process	positive regulation of response to stimulus	1.50E-07
GO:0044093	biological process	positive regulation of molecular function	0.020354587
GO:0048522	biological process	positive regulation of cellular process	3.36E-11
GO:0048518	biological process	positive regulation of biological process	1.34E-11
GO:0016310	biological process	phosphorylation	6.55E-10
GO:0006793	biological process	phosphorus metabolic process	9.70E-19
GO:0006796	biological process	phosphate-containing compound metabolic process	9.70E-19
GO:0016265	biological process	obsolete death	0.006437149
GO:0006397	biological process	mRNA processing	0.033147262
GO:0016071	biological process	mRNA metabolic process	0.012241772
GO:0005996	biological process	monosaccharide metabolic process	0.008865569
GO:0019941	biological process	modification-dependent protein catabolic process	0.020800796
GO:0043632	biological process	modification-dependent macromolecule catabolic process	0.020800796
GO:0008152	biological process	metabolic process	2.12E-59
GO:0043412	biological process	macromolecule modification	5.12E-15
GO:0043170	biological process	macromolecule metabolic process	1.65E-35
GO:0006629	biological process	lipid metabolic process	8.49E-06
GO:0006811	biological process	ion transport	6.80E-13
GO:0035556	biological process	intracellular signal transduction	3.04E-13
GO:0006954	biological process	inflammatory response	2.04E-05
GO:0002376	biological process	immune system process	0.011212838
GO:0006955	biological process	immune response	0.017093438
GO:0019318	biological process	hexose metabolic process	0.014826858
GO:0046483	biological process	heterocycle metabolic process	1.66E-21
GO:0007186	biological process	G-protein coupled receptor signaling pathway	0.009504177
GO:0045184	biological process	establishment of protein localization	1.23E-07
GO:0051234	biological process	establishment of localization	2.35E-34
GO:0006281	biological process	DNA repair	7.74E-15
GO:0016311	biological process	dephosphorylation	3.22E-11
GO:0006952	biological process	defense response	4.76E-06
GO:0006325	biological process	chromatin organization	1.33E-13
GO:0016568	biological process	chromatin modification	1.27E-08
GO:0051716	biological process	cellular response to stimulus	6.50E-40
GO:0006464	biological process	cellular protein modification process	8.53E-17
GO:0044267	biological process	cellular protein metabolic process	1.77E-14

GO:0009987	biological process	cellular process	5.66E-83
GO:0044237	biological process	cellular metabolic process	3.65E-40
GO:0044260	biological process	cellular macromolecule metabolic process	8.72E-28
GO:0044248	biological process	cellular catabolic process	0.004677883
GO:0007166	biological process	cell surface receptor signaling pathway	5.22E-10
GO:0008219	biological process	cell death	0.006437149
GO:0007155	biological process	cell adhesion	0.000276555
GO:0006812	biological process	cation transport	8.13E-08
GO:0009056	biological process	catabolic process	2.37E-05
GO:0008150	biological process	biological process	1.32E-144
GO:0065007	biological process	biological regulation	1.86E-71
GO:0022610	biological process	biological adhesion	0.000276555
GO:0006915	biological process	apoptotic process	0.007746723
GO:0044459	cellular component	plasma membrane part	8.33E-07
GO:0044422	cellular component	organelle part	1.60E-19
GO:0044451	cellular component	nucleoplasm part	0.000532619
GO:0044428	cellular component	nuclear part	4.10E-14
GO:0005739	cellular component	mitochondrion	0.034598683
GO:0044425	cellular component	membrane part	6.62E-24
GO:0016020	cellular component	membrane	5.95E-21
GO:0031224	cellular component	intrinsic component of membrane	7.73E-15
GO:0044446	cellular component	intracellular organelle part	5.76E-20
GO:0016021	cellular component	integral component of membrane	1.11E-14
GO:0005737	cellular component	cytoplasm	1.07E-14
GO:0044427	cellular component	chromosomal part	0.007851605
GO:0005575	cellular component	cellular component	4.78E-89
GO:0044464	cellular component	cell part	1.04E-64
GO:0019842	molecular function	vitamin binding	0.034019828
GO:0005215	molecular function	transporter activity	2.23E-19
GO:0022857	molecular function	transmembrane transporter activity	2.11E-12
GO:0004888	molecular function	transmembrane signaling receptor activity	6.15E-14
GO:0016772	molecular function	transferase activity, transferring phosphorus-containing groups	1.05E-14
GO:0016741	molecular function	transferase activity, transferring one-carbon groups	2.47E-08
GO:0016758	molecular function	transferase activity, transferring hexosyl groups	1.07E-08
GO:0016757	molecular function	transferase activity, transferring glycosyl groups	0.000543779
GO:0016747	molecular function	transferase activity, transferring acyl groups other than amino-acyl groups	0.001729132
GO:0016746	molecular function	transferase activity, transferring acyl groups	0.005602143
GO:0000989	molecular function	transcription factor activity, transcription factor binding	1.24E-06
GO:0000988	molecular function	transcription factor activity, protein binding	1.24E-06
GO:0003712	molecular function	transcription cofactor activity	1.75E-06

GO:0036459	molecular function	thiol-dependent ubiquitinyl hydrolase activity	1.02E-07
GO:0022892	molecular function	substrate-specific transporter activity	4.38E-16
GO:0022891	molecular function	substrate-specific transmembrane transporter activity	9.05E-13
GO:0022838	molecular function	substrate-specific channel activity	4.72E-05
GO:0036094	molecular function	small molecule binding	1.42E-28
GO:0038023	molecular function	signaling receptor activity	8.55E-16
GO:0004871	molecular function	signal transducer activity	1.15E-13
GO:0015291	molecular function	secondary active transmembrane transporter activity	0.012645954
GO:0008757	molecular function	S-adenosylmethionine-dependent methyltransferase activity	0.011391172
GO:0032553	molecular function	ribonucleotide binding	2.21E-25
GO:0004872	molecular function	receptor activity	2.10E-17
GO:0016462	molecular function	pyrophosphatase activity	1.75E-12
GO:0032555	molecular function	purine ribonucleotide binding	2.21E-25
GO:0035639	molecular function	purine ribonucleoside triphosphate binding	1.30E-25
GO:0017076	molecular function	purine nucleotide binding	2.21E-25
GO:0004725	molecular function	protein tyrosine phosphatase activity	8.22E-11
GO:0004713	molecular function	protein tyrosine kinase activity	0.035618962
GO:0004674	molecular function	protein serine/threonine kinase activity	0.000127064
GO:0004672	molecular function	protein kinase activity	1.62E-11
GO:0005515	molecular function	protein binding	2.84E-96
GO:0015079	molecular function	potassium ion transmembrane transporter activity	0.000836259
GO:0016773	molecular function	phosphotransferase activity, alcohol group as acceptor	4.06E-13
GO:0042578	molecular function	phosphoric ester hydrolase activity	1.40E-15
GO:0008081	molecular function	phosphoric diester hydrolase activity	5.62E-05
GO:0004721	molecular function	phosphoprotein phosphatase activity	1.78E-10
GO:0070011	molecular function	peptidase activity, acting on L-amino acid peptides	9.50E-13
GO:0008233	molecular function	peptidase activity	1.33E-11
GO:0022803	molecular function	passive transmembrane transporter activity	4.72E-05
GO:0016491	molecular function	oxidoreductase activity	0.001291082
GO:0097159	molecular function	organic cyclic compound binding	8.49E-99
GO:0016779	molecular function	nucleotid yltransferase activity	0.000464637
GO:0000166	molecular function	nucleotide binding	6.00E-28
GO:0017111	molecular function	nucleoside-triphosphatase activity	8.09E-12
GO:1901265	molecular function	nucleoside phosphate binding	6.00E-28
GO:0004518	molecular function	nuclease activity	0.00545605
GO:0003774	molecular function	motor activity	0.00169836
GO:0003674	molecular function	molecular function	1.76E-224
GO:0060089	molecular function	molecular transducer activity	1.15E-13

GO:0008168	molecular function	methyltransferase activity	0.000172775
GO:0046873	molecular function	metal ion transmembrane transporter activity	8.55E-05
GO:0016874	molecular function	ligase activity	4.30E-05
GO:0016301	molecular function	kinase activity	4.02E-13
GO:0015075	molecular function	ion transmembrane transporter activity	2.15E-07
GO:0005216	molecular function	ion channel activity	4.72E-05
GO:0022890	molecular function	inorganic cation transmembrane transporter activity	0.000242794
GO:0004553	molecular function	hydrolase activity, hydrolyzing O-glycosyl compounds	0.000212222
GO:0016818	molecular function	hydrolase activity, acting on acid anhydrides, in phosphorus-containing anhydrides	1.12E-12
GO:0016817	molecular function	hydrolase activity, acting on acid anhydrides	1.66E-12
GO:0016787	molecular function	hydrolase activity	1.37E-44
GO:1901363	molecular function	heterocyclic compound binding	8.49E-99
GO:0032561	molecular function	guanyl ribonucleotide binding	0.004941736
GO:0019001	molecular function	guanyl nucleotide binding	0.004941736
GO:0003924	molecular function	GTPase activity	0.001861745
GO:0005525	molecular function	GTP binding	0.012900568
GO:0004930	molecular function	G-protein coupled receptor activity	0.02060625
GO:0004175	molecular function	endopeptidase activity	3.73E-07
GO:0003677	molecular function	DNA binding	4.54E-45
GO:0015267	molecular function	channel activity	4.72E-05
GO:0008324	molecular function	cation transmembrane transporter activity	0.000297471
GO:0005261	molecular function	cation channel activity	0.001401391
GO:0003824	molecular function	catalytic activity	1.79E-73
GO:0005488	molecular function	binding	7.30E-190
GO:0016887	molecular function	ATPase activity	0.000290959
GO:0005524	molecular function	ATP binding	2.11E-24
GO:0032559	molecular function	adenyl ribonucleotide binding	7.93E-24
GO:0030554	molecular function	adenyl nucleotide binding	7.93E-24
GO:0022804	molecular function	active transmembrane transporter activity	0.015954346
GO:0003779	molecular function	actin binding	1.63E-06

Table S17. Distribution of SNVs in the Yangtze finless porpoise genome

Type (alphabetical order)	Count	Percent
5_prime_UTR_variant	1	0%
Downstream gene variant	112,290	4.45%
Intergenic	1,623,467	64.27%
Introns	649,368	25.71%
Missense variant	12,227	0.48%
Splice acceptor variant	80	0.00%
Splice donor variant	104	0.00%
Splice region variant	1,976	0.08%
Start lost	72	0.00%
Stop gained	201	0.01%
Stop lost	55	0.00%
Stop retained variant	9	0%
Synonymous variant	11,036	0.44%
Upstream gene variant	115,285	4.56%
**Total**	**2,526,171**	**100%**

Table S18.The distribution of InDels in the Yangtze finless porpoise genome

Location	Count
Intergenic	198,514
Upstream gene variant	14,690
Downstream gene variant	13,096
Intron	93,549
Exonic	
Frameshift	430
Inframe	139
**Total**	**320,821**
